# Global Transcriptome Analysis of the Peach (*Prunus persica*) in the Interaction System of Fruit–Chitosan–*Monilinia fructicola*

**DOI:** 10.3390/plants13050567

**Published:** 2024-02-20

**Authors:** Polina C. Tsalgatidou, Anastasia Boutsika, Anastasia G. Papageorgiou, Andreas Dalianis, Maria Michaliou, Michael Chatzidimopoulos, Costas Delis, Dimitrios I. Tsitsigiannis, Epaminondas Paplomatas, Antonios Zambounis

**Affiliations:** 1 Department of Agriculture, University of the Peloponnese, 24100 Kalamata, Greece; polina.tsalgatidou@go.uop.gr (P.C.T.); k.delis@uop.gr (C.D.); 2 Institute of Plant Breeding and Genetic Resources, ELGO-DEMETER, 57001 Thessaloniki, Greece; bouanastasia@outlook.com; 3 Laboratory of Plant Pathology, Department of Crop Science, Agricultural University of Athens, 11855 Athens, Greece; anastasia.papageorgiou.g@gmail.com (A.G.P.); dimtsi@aua.gr (D.I.T.); epaplom@aua.gr (E.P.); 4 Laboratory of Vegetable Crops, Institute of Olive Tree, Subtropical Crops and Viticulture, ELGO-DEMETER, 71307 Heraklion, Greece; andreas_dalianis@hotmail.com (A.D.); mariamichaliou00@gmail.com (M.M.); 5 Department of Agriculture, International Hellenic University, 57400 Thessaloniki, Greece; mxatzid@agro.ihu.gr

**Keywords:** chitosan, *Monilinia fructicola*, priming, peach fruit, cell wall, transcriptomics, elicitor, plant–microbe interactions, antimicrobial compounds, plant immunity

## Abstract

The peach (*Prunus persica* L.) is one of the most important stone-fruit crops worldwide. Nevertheless, successful peach fruit production is seriously reduced by losses due to *Monilinia fructicola* the causal agent of brown rot. Chitosan has a broad spectrum of antimicrobial properties and may also act as an elicitor that activate defense responses in plants. As little is known about the elicitation potential of chitosan in peach fruits and its impact at their transcriptional-level profiles, the aim of this study was to uncover using RNA-seq the induced responses regulated by the action of chitosan in fruit–chitosan–*M. fructicola* interaction. Samples were obtained from fruits treated with chitosan or inoculated with *M. fructicola*, as well from fruits pre-treated with chitosan and thereafter inoculated with the fungus. Chitosan was found to delay the postharvest decay of fruits, and expression profiles showed that its defense-priming effects were mainly evident after the pathogen challenge, driven particularly by modulations of differentially expressed genes (DEGs) related to cell-wall modifications, pathogen perception, and signal transduction, preventing the spread of fungus. In contrast, as the compatible interaction of fruits with *M. fructicola* was challenged, a shift towards defense responses was triggered with a delay, which was insufficient to limit fungal expansion, whereas DEGs involved in particular processes have facilitated early pathogen colonization. Physiological indicators of peach fruits were also measured. Additionally, expression profiles of particular *M. fructicola* genes highlight the direct antimicrobial activity of chitosan against the fungus. Overall, the results clarify the possible mechanisms of chitosan-mediated tolerance to *M. fructicola* and set new foundations for the potential employment of chitosan in the control of brown rot in peaches.

## 1. Introduction

Brown rot caused by the fungal pathogen *Monilinia fructicola* is an important peach fruit disease responsible for major postharvest production losses [1]. Crop-protection-management strategies against this necrotrophic pathogen rely mainly on conventional fungicides [2]. However, the rapid pathogen evolution, the legislation limitations on the usage of these fungicides, combined with the increasing development of pathogen resistance on major chemical classes of fungicides, have raised the need to develop alternative and environmentally friendly strategies towards a more sustainable and resilient disease management of peach brown rot [3]. 

Nowadays, exploiting the resistance inducers is a novel strategy to elicit defense responses in fruits against pathogen infections [4]. Chitosan (poly β-(1 → 4) N-acetyl-d-glucosamine), a non-toxic high-molecular-weight polysaccharide produced by the deacetylation of chitin, has emerged as a promising substitute for synthetic chemical fungicides for the efficient control of fruit diseases [5,6]. Its broad-spectrum direct antimicrobial activity has been recorded against several fungal plant pathogens including *M. fructicola* [7,8]. Specifically, chitosan not only inhibits mycelial growth and spore germination, but it also induces pronounced morphological changes and structural alterations of hyphae, damages cell surface architecture and protein biosynthesis, and significantly affects fungal genes’ expression profiles [9,10,11,12]. 

Despite its direct antifungal activity, chitosan is also known to induce systemic resistance in plants, acting as an exogenous plant defense elicitor [13]. Thus, postharvest-coating applications of chitosan in fruits activate defense mechanisms leading to physiological and biochemical changes, and suppression of many diseases including gray mold caused by *Botrytis cinerea* in grapes and strawberries [14], green mold caused by *Penicilium digitatum* in citrus [15], anthracnose disease caused by *Colletotrichum* spp. in papaya [16], and blue mold caused by *Penicilium expansum* in kiwifruit [17]. These defense responses are usually highly coordinated, integrating various cell wall-modification processes, inducing specific pathogenesis-related (PR) proteins, and synthesizing secondary metabolites with antimicrobial activity such as polyphenolic compounds, flavonoids, and phytoalexins [18,19]. Therefore, chitosan as a defense-priming agent can stimulate fine-tuned immunity transcriptomic responses that act at specific defense levels and vary depending on the type of plant–pathogen interaction [20]. For instance, cell-wall fortification and effective production of reactive oxygen species (ROS) has been used as a marker for the expression of priming responses [21]. 

Though several studies have demonstrated the effects of chitosan on controlling or delaying peach brown rot disease after postharvest formulation [22,23,24], the molecular mechanisms behind chitosan application are not completely understood for peach fruits, and no transcriptomic study has yet been conducted to evaluate the impact of chitosan on *M. fructicola* infection. Such transcriptome studies using an RNA-seq approach have been previously reported in chitosan-treated sweet orange [18], strawberry [4], and avocado [25] fruits. Furthermore, large-scale gene expression analysis of grape fruits after the postharvest application of chitosan revealed changes in their transcriptional profile providing tolerance to *B. cinerea* [26]. Target genes associated with plant-regulating signaling pathways, pathogenesis-related proteins, cell-wall-degrading enzymes, and the phenylpropanoid pathway were upregulated after postharvest chitosan application [16,17]. 

The aim of the present study was to explore the global transcriptional profile of peach fruits treated with chitosan in the presence or absence of *M. fructicola*, taking also into account the transcriptomics changes in gene expression patterns upon a compatible interaction. Our study allows for deciphering the impact of chitosan on the induction of defense-related pathways to *M. fructicola*, and we report the differentially expressed genes (DEGs) that could be involved in the tolerance induced by this elicitor in peach fruit. Based on this study, we discuss putative genes that are driving the expression of priming mechanisms through which chitosan reduces the severity of brown rot in peach fruits. We also identified a set of highly induced *M. fructicola* genes that could aid in elucidating the fungal genes involved in peach pathogenesis, as well addressing whether chitosan, besides triggering defense priming, exhibits direct antimicrobial activity against the pathogen. To the best of our knowledge, this is the first report deciphering the large-scale transcriptional impact of chitosan when it was applied alone or as a pre-fungal inoculation treatment, in comparison with the impact of the fungal infection alone in the peach–*M. fructicola* interaction. We also evaluated the impact of a well-defined chitosan to suppress brown rot disease in peach fruit after postharvest-coating formulation, whereas physiological indicators were monitored in the chitosan-mediated control of the disease. Our findings, along with the outlined characteristics of chitosan settle this substance as a suitable component in the integrated disease management of brown rot in peach fruits.

## 2. Results

### 2.1. Disease Reduction in Peach Fruits Treated with Chitosan

**Figure 1:**
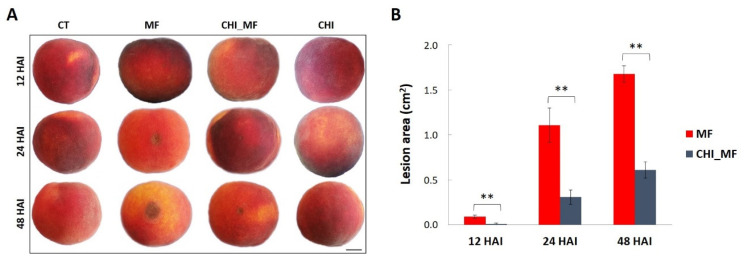
Disease severity of peach fruits against *M. fructicola*. (**A**) Brown rot symptoms of peach fruits with (CHI_MF) and without (MF) chitosan pre-treatment at 12, 24, and 48 HAI. Peach fruits treated only with chitosan (CHI) and untreated–mock-inoculated (CT) fruits were used as control groups (scale bar: 30 mm). (**B**) Lesion area (cm^2^) on untreated peach fruits after inoculation with *M. fructicola* (MF) and on peach fruits inoculated with *M. fructicola* after chitosan pre-treatment (CHI_MF). Bars represent the mean of 3 biological replicates ± standard deviation after *t*-test analysis. Asterisks indicate statistically significant differences between MF and CHI_MF treatments at each inoculation time point (*p* < 0.01).

All *M. fructicola*-inoculated fruits (MF treatment) developed visual brown rot necrotic lesions around the inoculation sites within 24 h after inoculation (HAI). These fruits showed more drastic symptoms in terms of growth and expansion of the pathogen than fruits pre-treated with chitosan prior to their inoculation with *M. fructicola* (CHI_MF treatment). In that case, the pathogen was significantly spread with a delay, resulting in less deterioration of quality and maintaining a lower expansion rate through the 48 HAI period (Figure 1A). The lesion area in both MF and CHI_MF treatments increased progressively around the inoculation sites throughout time, covering approximately 1.7 and 0.6 square centimeters of fruits at 48 HAI, respectively (Figure 1B). Peaches treated only with chitosan (CHI) and untreated control fruits (CT) were also used. These results indicate that peach fruit tolerance against *M. fructicola* increases rapidly prior to chitosan application, highlighting the inhibitory effect of chitosan on brown rot disease development on peach fruit.

### 2.2. Physiological Alterations of Peach Fruits

**Figure 2:**
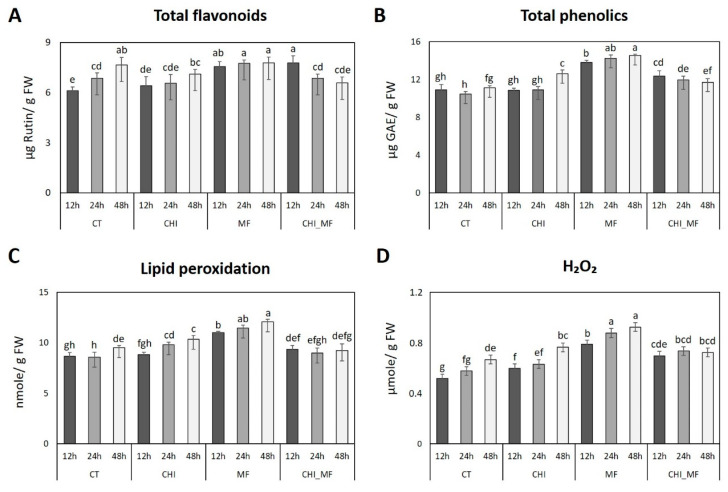
Determination of physiological indexes of peach fruits with chitosan (CHI), *M. fructicola* (MF), both chitosan and *M. fructicola* (CHI_MF), and untreated–mock-inoculated (CT) treatments across three time points. (**A**) Total flavonoids, (**B**) total phenolics, (**C**) lipid peroxidation (thiobarbituric acid-reactive substances; TBARS), and (**D**) hydrogen peroxide (H_2_O_2_). Bars indicate the mean values of three biological replicates ± standard deviations. A statistical analysis was performed using one-way ANOVA followed by Tukey’s multiple comparison post hoc test (*p* < 0.05). Different letters indicate statistical differences among treatments at all time points.

An immediate response of plants to biotic stress is the production of antioxidant secondary metabolites like phenolic and flavonoid compounds, in order to reduce lipid peroxidation and H_2_O_2_ accumulation, as a response to oxidative stress. The level of flavonoid compounds in CT and CHI treatments increased progressively with time, reaching the highest amount at 48 HAI. However, in MF treatment the total flavonoids were higher than in other treatments through all the sampling (Figure 2A), reaching the most significant, highest amount at 48 HAI. In CHI_MF treatment, the amount of total flavonoids reached its highest value at 12 HAI and declined progressively over time to 6.6 μg g^−1^ FW. 

The amount of total phenolics in peach fruits inoculated only with *M. fructicola* was significantly higher than that in any other treatment, at any time point (Figure 2B). Specifically, total phenolic compounds in the MF treatment significantly (*p* = 0.05) increased from 13.8 to 14.6 μg g^−1^ FW (fresh weight), reaching the highest amount at 48 HAI. CT fruits presented the lowest amount of total phenolic compounds compared to other treatments. Although the level of total phenolics in CHI treatment at 12 and 24 HAI showed no significant difference with CT treatment, at 48 HAI total phenolic compounds significantly increased reaching 12.6 μg g^−1^ FW. The concentration of phenolic compounds in the CHI_MF treatment exhibited a significant decrease in comparison to the MF treatment at all time points, albeit remaining higher than that of the CT treatment.

As depicted in Figure 2C, MF treatment significantly triggered lipid peroxidation of peach fruits, showing much higher thiobarbituric acid-reactive substance (TBARS) levels compared to any other treatment even at 12 HAI. High levels of TBRAS in peach fruits treated with *M. fructicola* indicates an immediate oxidative response to the fungal infection. TBRAS levels of MF treatment increased progressively reaching the highest amount at 48 HAI (12.1 nmole g^−1^ FW). Both CT and CHI_MF treatments presented similar levels of TBARS, being much lower compared to MF treatment at any time point. CHI treatment triggered also lipid peroxidation of peach fruit, in a time-dependent manner although keeping a lower level of TBRAS compared to MF treatment.

Hydrogen peroxide (H_2_O_2_) levels were correlated to TBARS levels in any treatment (Figure 2D). As in lipid peroxidation assay, fruits in MF treatment reached higher level of H_2_O_2_ in a time-dependent manner, followed by CHI and CHI_MF treatment, whereas in CT treatment the H_2_O_2_ level was significantly low.

### 2.3. Data Overview of RNA-Seq Analysis and Mapping

To contribute to the understanding of how CHI treatment promotes changes in transcript expression in peach fruits, transcriptional profiles of fruit samples treated with the elicitor (CHI treatment), inoculated with the fungus (MF treatment), chitosan pre-treated and inoculated with the fungus (CHI_MF treatment), and those that were untreated–mock inoculated (CT treatment) were generated employing RNA-seq. Thus, a total of 36 fruit samples were subjected to RNA-seq analysis harboring three biological replicates for each treatment across the three different set times after inoculation (12, 24 and 48 HAI). Each sample produced 8.27 G data on average. A total of 1,969,927,470 high-quality pair-end reads were generated and approximately 91.58% of reads could be mapped uniquely to the reference peach genome (Table S1). Furthermore, only a small percentage of the clean reads compared with the reads assigned to peach genomic assembly could be mapped onto a limited number of *M. fructicola* genes in cases of MF and CHI_MF treatments, mainly at 48 HAI (Table S2).

### 2.4. Differential Gene Expression Profiles of Peach Fruits

**Figure 3:**
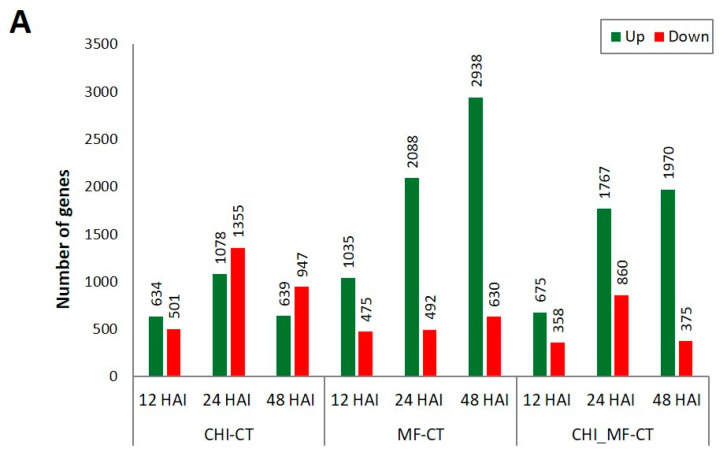

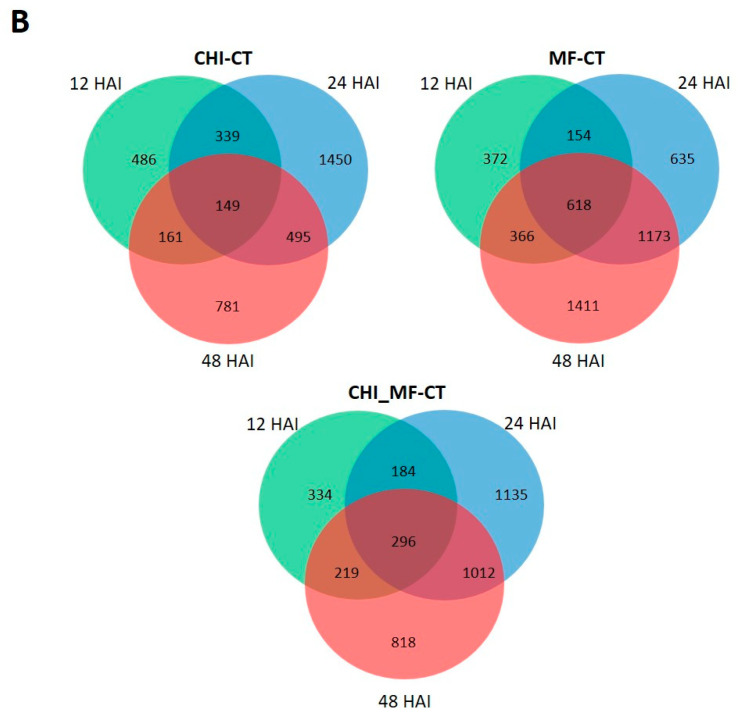
(**A**) Gene number of up/downregulated DEGs among the three different comparison groups (CHI–CT, MF–CT, CHI_MF–CT) at 12, 24, and 48 HAI. (**B**) Venn diagram showing DEGs commonly regulated across the three time points for each comparison group; CHI: chitosan-treated fruits, MF: *M. fructicola*-inoculated fruits, CHI_MF: chitosan-pre-treated and *M. fructicola*-inoculated fruits, CT: untreated–mock-inoculated fruits.

RNA-seq data of all treatments were allocated into three comparison groups, namely CHI–CT, MF–CT, and CHI_MF–CT. The DEGs between CHI, MF, and CHI_MF fruit samples versus mock-inoculated samples (CT) were assigned with a threshold of log2fold change ≥ 1 across the three time points (Table S3). A histogram counted the number of up/downregulated DEGs in the three comparison groups (Figure 3A), and Venn diagrams (Figure 3B) were generated for all comparison groups showing the number of DEGs and the overlap between them at each time point. Moreover, volcano plots visually illustrate the correlation between the number of up/downregulated DEGs across groups (Figure S1), whereas Venn diagrams show DEGs commonly regulated across the three time points across the four treatments (Figure S2). Our results revealed that the highest number of DEGs at 12 and 48 HAI were observed for the MF-CT comparison group, whilst for this group at 48 HAI the DEGs were the most abundant among all comparison groups across the three time points with the highest proportion of upregulated genes. Furthermore, all DEGs in the MF-CT group were progressively upregulated across time points. However, at 24 HAI the highest levels of DEGs, mostly upregulated, were recorded for the CHI_MF-CT comparison group which indicates that chitosan may have a stronger effect on this time point after inoculation with *M. fructicola*. In accordance with the CHI-CT comparison group, the highest DEGs levels were observed also at 24 HAI, followed by the DEGs at 48 HAI, which were mainly downregulated at both time points.

### 2.5. Classification of DEGs upon Gene Ontology Categorization

DEGs were assigned to significant functional annotations upon GO term enrichment analysis and classified accordingly to their molecular function (MF), cellular component (CC), and biological process (BP) (Figure S3). For the CHI-CT comparison group, the GO term “oxidoreductase activity” corresponding to molecular functions was a constitutively significant enriched group among all time points. Furthermore, the GO term “chitinase activity” was significantly enriched only at 48 HAI, while the cellular component GO term “cell wall” was only enriched early at 12 and 24 HAI. The GO terms associated with photosynthesis, such as “thylakoid” and “photosynthetic membrane”, were significantly upregulated at 24 HAI, whereas both were downregulated at the late time point. In terms of biological processes, “defense response”, “response to biotic stimulus”, and “response to stress” terms were all significantly suppressed at 12 HAI, whereas, conversely, at this time point “fatty acid biosynthetic process” and “lipid biosynthetic process” terms were significantly upregulated. Finally, GO terms related with chitin catabolic and metabolic processes were significantly upregulated at 48 HAI (Figure S3A).

For the MF-CT comparison group, DEGs that were upregulated were associated with the enriched terms “defense response” and “response to biotic stimulus” which were only recorded with a delay and gradually from 24 to 48 HAI. However, such unravelling defense responses were not effective, since the fruits have severe brown rot lesions at these time points. A similar enrichment pattern was revealed upon molecular functions such as on “transcription regulator activity” and “pattern binding” GO terms (Figure S3B).

For the MF_CHI-CT comparison group, at 12 HAI genes associated with molecular functions such as “fatty acid biosynthetic process” and “fatty acid metabolic process” were mostly upregulated, whereas “defense response” and “response to biotic stimulus” only increased with a delay, as in the MF-CT comparison group. DEGs associated with photosynthesis-related GO terms were upregulated mostly at 24 HAI. Furthermore, DEGs associated with molecular functions such as “oxidoreductase activity”, “transcription regulator activity”, and “cofactor binding” were mostly upregulated at 12 HAI, followed by a progressive upregulation of DEGs associated with similar GO terms from 24 up to 48 HAI. Notably, DEGs associated with “polysaccharide binding” and “chitinase activity” were mostly upregulated at 48 HAI (Figure S3C).

### 2.6. KEGG Metabolic Pathway Enrichment Analysis

**Figure 4:**
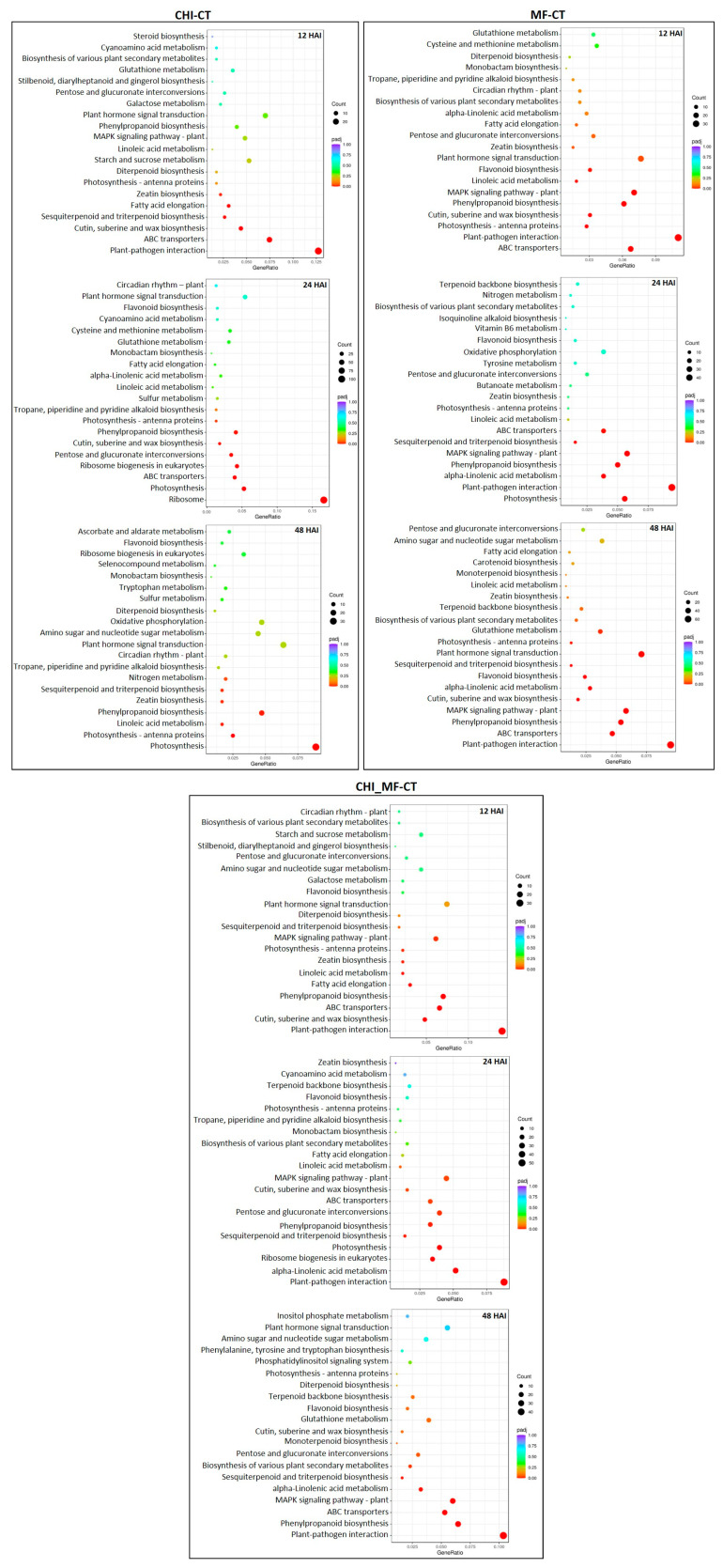
Classification of the peach fruits DEGs in KEGG pathways across the three comparison groups, after chitosan treatment or *M. fructicola* inoculation, either individually or in combination, at three time points (12, 24 and 48 HAI). The counts of the DEGs being annotated in the corresponding pathways are depicted.

To further analyze the metabolic pathways and the biological functions of DEGs in the presence of *M. fructicola* (MF) and chitosan (CHI), individually or in combination (CHI_MF), we performed a KEGG functional enrichment analysis at each time point. DEGs for all comparison groups were further assigned to 20 of the most enriched pathway entries to identify the involved KEGG pathways in peach transcriptomes (Figure 4). For the CHI-CT group, significant enrichment of a number of DEGs related to “plant pathogen interaction” was remarkably observed at 12 HAI, along with the upregulation of several DEGs related to pathways including “ABC transporters”, “cutin, suberine, and wax biosynthesis”, “sesquiterpenoid and triterpenoid biosynthesis” and “fatty acid elongation”. For the same group, significant upregulation of a large number of DEGs at 24 HAI related to photosynthesis-associated pathways was observed, whereas at 48 HAI these pathways were significantly suppressed. The defense-related pathway “phenylpropanoid biosynthesis” involved in secondary metabolites’ biosynthesis, as well as pathways associated with primary metabolism were induced at 24 and 48 HAI.

For the MF-CT group, the “plant–pathogen interaction” pathway was the highest enriched category, with the number of related DEGs gradually increasing from 12 HAI to the late time points, as did DEGs related to “phenylpropanoid biosynthesis”. Moreover, the “ABC transporters” pathway was constitutively enriched at all time points. Progressively, several defense-related pathways were mainly enriched at 48 HAI such as the “MAPK signaling pathway”, “flavonoid biosynthesis”, “sesquiterpenoid and triterpenoid biosynthesis”, “alpha-Linolenic acid metabolism”, and “plant hormone signal transduction” (Figure 4). 

Finally, in MF_CHI-CT as in the MF-CT group, “plant–pathogen interaction” was the highest upregulated pathway with the number of related DEGs being increased from 12 HAI up to the late time point. Nevertheless, the number of relevant DEGs was slightly lower, with induction rates that were less pronounced when compared to the MF-CT group at the respective time points. Pathways related to the induction of defense responses, such as “ABC transporters”, “phenylpropanoid biosynthesis”, “sesquiterpenoid and triterpenoid biosynthesis”, “alpha-Linolenic acid metabolism”, and “MAPK signaling pathway” were constitutively enriched at all time points, but mostly at 48 HAI. While photosynthesis-related pathways were significantly upregulated at 12 and 24 HAI, the “fatty acid elongation” pathway was upregulated early at 12 HAI, similar to the CHI-CT group (Figure 4).

### 2.7. Peach DEGs Involved in Cell-Wall Degradation and Modification

**Figure 5:**
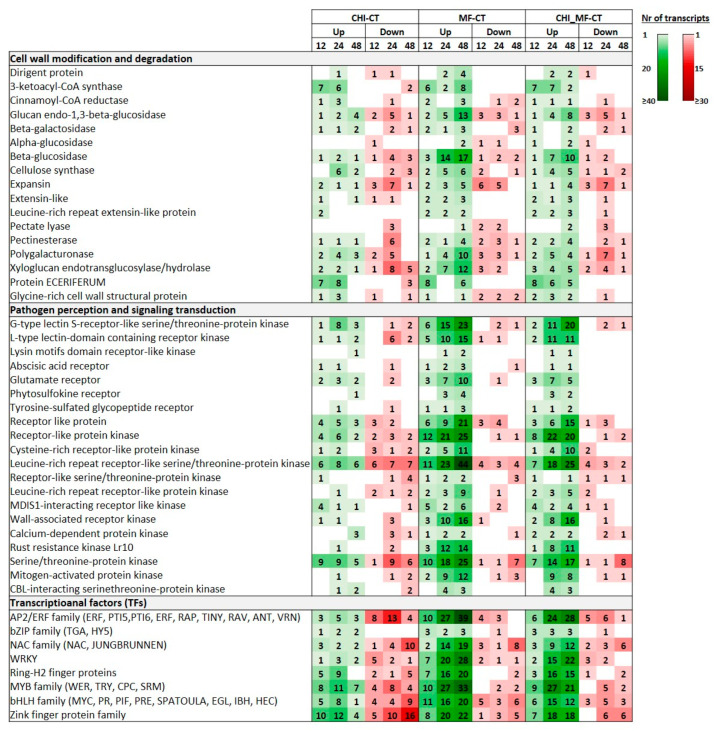

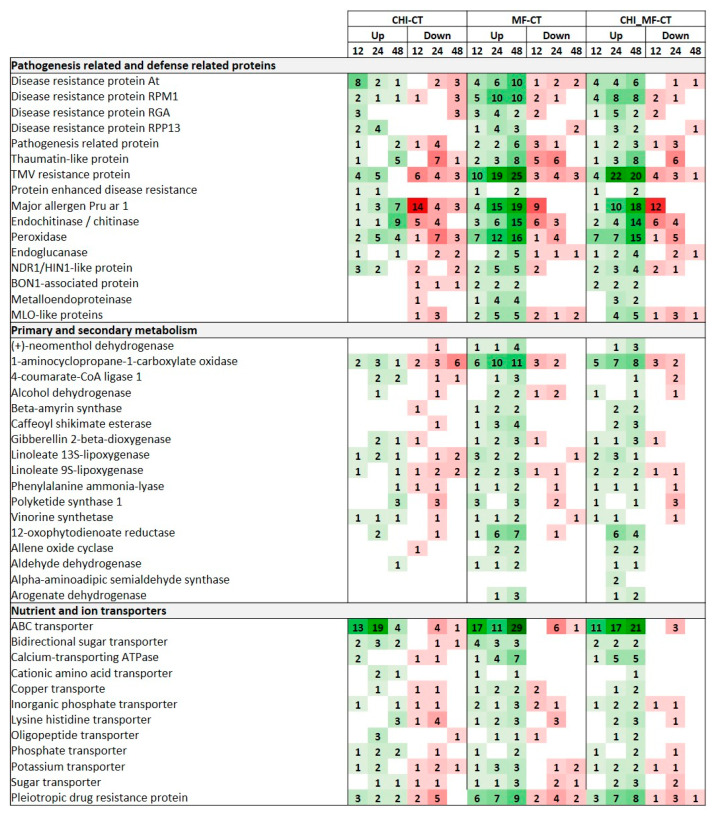
Selection of key DEGs upregulated (Up) and downregulated (Down) in peach fruit after application of chitosan (CHI), inoculation with *M. fructicola* (MF), or in combination (CHI_MF) in comparison to control (CT) treatment. For each gene category, the numbers of differentially expressed transcripts are reported.

Notably, DEGs encoding 3-ketoacyl-CoA synthase (KCSs) that was involved in cell wall-fortification processes were constitutively upregulated both at the CHI-CT and CHI_MF-CT comparison groups at 12 and 24 HAI, whereas such genes were also upregulated at the MF-CT group at 48 HAI. For the CHI-CT group, genes encoding cellulose synthase (*CesA*) and cinnamoyl-CoA reductase (CCR) were mainly upregulated at 24 HAI; such genes were also induced in the other two groups at 24 HAI but were lower in their numbers. Remarkably, eceriferum (*CER*) genes were constitutively upregulated only in the CHI_MF-CT group across the 48 period after *M. fructicola* inoculation, while they were also upregulated in the CHI-CT at 12 and 24 HAI. Furthermore, at the CHI-CT group the expression patterns of the majority of DEGs encoding members of expansin (EXP), pectate lyase (PL), and pectinesterase (PME), that promote susceptibility, were mostly suppressed at 24 HAI. Similar expression profiles were recorded in the CHI_MF-CT group for this time point, whilst by contrast such DEGs were mainly upregulated at the MF-CT group at 48 HAI. Numerous DEGs encoding glucan endo-1,3-beta-glucosidase and β-glucosidase were almost constitutively upregulated in the MF-CT group, however, to a considerably lesser extent in the CHI_MF-CT group (24 and 48 HAI). Furthermore, polygalacturonase (*PGs*) genes were significantly upregulated at 48 HAI mainly in the MF-CT group. In contrast, regarding xyloglucan endotransglucosylase/hydrolase (XTH)-encoding genes, their upregulation was more evident in MF-CT compared to the CHI_MF-CT group. In the MF-CT group, a few dirigent (DIR) protein DEGs were significantly upregulated at 48 HAI, whereas the activation of glycine-rich protein (*GRP*) genes was pronounced both at the CHI-CT and CHI_MF-CT groups. Finally, both at the MF-CT and CHI_MF-CT groups the expression patterns at 48 HAI revealed the constitutive upregulation of extensin (*EXT*) and leucine-rich repeat containing *EXTs* genes (*LRX*) (Figure 5; Table S4). 

### 2.8. Peach DEGs Involved in Pathogen Perception and Signaling Transduction

An abundant inventory of pathogen recognition receptor (*PRR*)-encoding genes was significantly induced upon *M. fructicola* infection, including various types of receptor-like kinases (RLKs) and receptor-like proteins (RLPs), mainly at 24 and 48 HAI, as revealed both for the MF-CT and CHI_MF-CT groups. The observed expression patterns suggest that such DEGs were more prominently induced in the MF-CT group rather than in groups subjected to chitosan treatment. Thus, apart from the RLKs, several DEGs encoding receptors containing lectin domains (LecRKs) such as G-type lectin S-receptor-like serine/threonine-protein kinases (GsSRKs) and L-type lectin-domain containing receptor kinase (L-type LecRLKs), or lysin motifs (LysM RLK) were highly upregulated mainly at 48 HAI in both the MF-CT and CHI_MF-CT groups. Among the other DEGs involved in pathogen perception that were mostly progressively induced at late time points upon *M. fructicola* infection, particularly in the MF-CT group, were those encoding various types of RLPs such as cysteine-rich receptor-like protein kinases (CRKs), glutamate receptors (GRs), wall-associated receptor kinases (WAKs), and phytosulfokine receptors (PSKRs). It is worth mentioning that abscisic acid (ABA) receptors were mostly upregulated for the MF-CT group at 48 HAI. Similarly, DEGs encoding RLKs containing LRR domains (LRR-RLKs) were progressively upregulated at both MF-CT and CHI_MF-CT groups, and at 48 HAI having a higher number of activated DEGs associated with pathogen perception. In parallel, DEGs encoding rust resistance kinase (RRK), serine/threonine-protein kinase (STPK), mitogen-activated protein kinase (MAPK), CBL-interacting serine/threonine-protein kinases (CIPKs), and calcium-dependent protein kinase (CDPK) were induced mostly at late stages of infection with a higher expression ratio in the MF-CT group compared to the CHI_MF-CT group. All DEGs involved in pathogen perception in the CHI-CT group were induced to significantly lower levels in comparison to the other groups, whereas they were mainly induced at 12 and 24 HAI (Figure 5; Table S5).

### 2.9. Peach DEGs Encoding TFs

Several TF-encoding genes belonging to different families were strongly induced in peach fruits infected with *M. fructicola* (MF-CT, CHI_MF-CT comparison groups), mostly at 24 HAI and 48 HAI. Specifically, DEGs encoding AP2/ERF, NAC, WRKY, MYB, bHLH, ZFP, and Ring-H2 finger TFs were constitutively and progressively over-expressed at three time points, reaching, in most cases, their highest number at 48 HAI. These *TFs* were less induced for the CHI-CT group. Thus, for example, 39 and 28 *AP2*/*ERFs* were upregulated in the MF-CT and CHI_MF-CT groups at 48 HAI, respectively, whilst a slightly lower expression ratio was recorded for them in the CHI_MF-CT compared to the MF-CT group; the induction of *AP2*/*ERFs* at CHI-CT was less evident, and notably at 24 HAI the majority of them were suppressed. However, in the CHI-CT group, four members of ZFPs containing the CCCH domain were constitutively upregulated at an early time point, which is in contrast with the other groups (Figure 5; Table S6). 

### 2.10. Peach DEGs Encoding Pathogenesis-Related and Defense Proteins

Our RNA-seq data indicated that, in the CHI-CT group, DEGs encoding types of PR proteins were only spatially induced across the time period. This expression profile was only slightly altered in the case of endochitinase and thaumatin encoding DEGs, with nine and five members being upregulated at 48 HAI, respectively, as well for DEGs encoding Pru ar (major allergen Pru ar 1) with 14 members being downregulated at 12 HAI and seven members being upregulated at 48 HAI. On the contrary, in the MF-CT group a plethora of various *PRs* were upregulated, particularly at 48 HAI, such as DEGs encoding endochitinase (PR-3), endoglucanase (PR-2), peroxidase (PR-9), metalloendoproteinase 2-MMP (PR-10), Pru ar (PR-10), and thaumatin (PR-5). Furthermore, DEGs encoding members of disease-resistance proteins of the NHL (NDR1/HIN1), RPM1, RPP13, MLO families, and BON1-associated proteins (BAPs) were also highly upregulated at 48 HAI. In the CHI_MF-CT group, the expression patterns were similar to those of DEGs of the MF-CT group, whilst slightly less DEGs were induced along with lower induction rates (Figure 5; Table S7). 

### 2.11. Peach DEGs Involved in Secondary and Primary Metabolism

At the CHI-CT group quite a few DEGs related to primary and secondary metabolites’ biosynthesis were induced in comparison with other groups. On the other hand, in the MF-CT group several DEGs involved in the induction of phenylpropanoid biosynthesis, which was one the most enriched pathways across all time points, were upregulated mainly at a late time point. Phenylpropanoid biosynthesis is the key procress in the biosynthesis of phenols, along with the key enzyme phenylalanine ammonia-lyase (PAL), whereas two *PAL* genes were highly upregulated at 48 HAI. Other DEGs encoding precursors of phenol biosynthesis (tyrosine and tryptophan) were progressively induced from 24 HAI and mainly at 48 HAI. Similarly, DEGs involved in ethylene biosynthesis (like 1-aminocyclopropane-1-carboxylate oxidase, ACO), flavonoid biosynthesis (like polyketide synthase, PKS), sesquiterpenoid and triterpenoid biosynthesis, and terpenoid backbone biosynthesis were also highly upregulated, mainly at 48 HAI. Furthermore, the pathway of “alpha-linolenic acid metabolism” involved in primary metabolism was enriched mostly through the constitutive upregulation of genes encoding 12-oxophytodienoate reductase (OPR) and allene oxide cyclase (AOC) at 24 and 48 HAI. In the CHI_MF-CT group, similar DEGs were also induced mainly at a late time point, however, to a lesser degree in comparison to the MF-CT group (Figure 5; Table S8). 

### 2.12. Peach DEGs Encoding Nutrient and Ion Transporters

Several sorts of transporter encoding DEGs were significantly induced in the CHI-CT group at early time points and mainly at 24 HAI, such as the ABC transporters. In contrast, in the other two groups involving *M. fructicola* inoculations, the majority of them were upregulated mainly at 48 HAI. Among the ABC transporters, DEGs of the pleiotropic-drug-resistance (*PDR*) gene family were also induced at both the MF-CT and CHI_MF-CT groups at late time points. In addition, various calcium-transporting ATPases along with potassium, oligopeptide, lysine histidine, and phosphate transporters were also induced and mainly upregulated in the MF-CT and CHI_MF-CT groups, mostly at 24 and 48 HAI. Notably, the induction of DEGs was of a lower expression rate in the CHI_MF-CT compared to MF-CT group, as for example is the case for DEGs encoding lysine histidine transporters (LHTs) at 48 HAI (Figure 5; Table S9).

### 2.13. Monilinia Fructicola-Expressed Genes upon Inoculation in Peach Fruit

A closer examination of the most abundant and highly detected genes involved in *M. fructicola* growth and pathogenesis was conducted both in MF and CHI_MF treatments. In the absence of chitosan pre-treatment, a large number of reads were mapped to *M. fructicola* genes where most of the genes related to virulence were expressed at 12 HAI, but were mostly abundant at 48 HAI (Table S2). We detected several genes encoding hydrolytic or carbohydrate-active enzymes (CAZymes) that were highly expressed, alongside a single gene encoding a polygalacturonase (*PG1*/*EYC84_010610*), which presented the highest read counts upon infection. Several genes encoding a repertoire of different CAZyme classes were detected, related to plant cell-wall disassembly, including different families of glycoside hydrolases (GH5, GH17, GH28, GH43, GH45, GH53), carbohydrate esterases (CE8, CE12, CE16), and glycosyltransferases (GT2, GT39, GT66). The largest number of predicted CAZymes belonged to the pectin-degrading GH28 family of polygalacturonases which were highly enriched across all time points. Furthermore, two genes encoding the pectin-degrading enzyme pectin-methyl esterase (PME) (*EYC84_003200* and *EYC84_008212*), one gene encoding pectin lyase (PNL) (*EYC84_007082*), one gene encoding family 2 peroxidase (*EYC84_007266*), along with three genes encoding serine (*EYC84_008408*), aspartate (*EYC84_008126*), and acid (*EYC84_011533*) proteases were significantly abundant, mostly at 48 HAI. We further detected several highly expressed genes corresponding to oxidoreductase activity and encoding aldehyde dehydrogenase, acetylesterase, formate dehydrogenase, zinc-binding alcohol dehydrogenase, GMC oxidoreductase, and the AhpC/TSA family protein. Among the genes encoding effector proteins, we detected a gene (*EYC84_002901*) encoding the effector cerato-platanin (CP), mostly expressed at 48 HAI, whereas one gene (*EYC84_009186*) putatively encoding a necrosis and ethylene-inducing protein (NAP) was highly abundant across all time points. Moreover, we detected a gene (*EYC84_001382*) encoding a putative effector protein with a CFEM domain which was highly enriched at 12 HAI and gradually reduced its expression over time. 

By contrast, in CHI_MF treatment, most of the genes related to pathogenesis, sporulation, cell-membrane homeostasis, and ribosome biogenesis of *M. fructicola* were expressed at significantly low levels or were not even detected at any time point (Table S2). Notably, genes encoding several CAZymes, as well as genes encoding the AhpC/TSA family protein and the effector cerato-platanin protein, were not detected earlier than 24 HAI, recording a lower expression compared to the MF-CT group. *EYC84_007082* gene encoding PNL, *EYC84_004053* encoding GT2, and *EYC84_007266* encoding family 2 peroxidase were expressed at 48 HAI. Genes required for sporulation (*EYC84_000112*, *EYC84_002048* and *EYC84_003170*), as well as many genes encoding proteins corresponding to ribosome biogenesis such as urb1 (*EYC84_000100*), tsr1 (*EYC84_001134*), YTM1 (*EYC84_004958*), ALB1 (*EYC84_007576*), erb1 (*EYC84_010071*), Gar2 (*EYC84_005843*), and a GTP-binding ribosome biogenesis protein (*EYC84_003331*) were not expressed at any time point. Moreover, the induction of genes associated with pathways related to cell-membrane homeostasis was also disrupted compared to MF treatment. Several genes involved in glycerophospholipid metabolism, steroid biosynthesis and ergosterol biosynthesis processes were also not detected upon chitosan treatment. Overall, these results highlight the direct antimicrobial activity of chitosan on *M. fructicola*. A heat map of the hierarchical clustering of *M. fructicola* expressed genes based on the average counts of normalized reads among MF and CHI_MF treatments is shown in Figure S4.

### 2.14. Validation of Peach RNA-Seq Data Using qRT-PCR

The log2fold change values of RNA-seq analysis were validated using a quantitative real-time PCR (qRT-PCR) assay. A set of nine DEGs were randomly selected to validate the results of RNA-seq. All genes showed similar expression patterns to those of the RNA-seq data (Figure S5).

## 3. Discussion

Peach fruit is climacteric and has a short postharvest life with a high susceptibility to pathogens. Particularly, brown rot caused by the necrotrophic fungus *M. fructicola* is a major disease for peach fruit [24]. Natural resistance inducers have been proposed as an alternative and innovative approach for efficient disease control in crops [27]. Among them, chitosan, a versatile compound, is known, even at very low concentrations, to show direct antifungal activity and trigger, as a non-microbial elicitor, the defense mechanisms in plants that are challenged by pathogens [11,24,25]. Therefore, due to the dual nature of its action, chitosan is an autocidal fungal pathogen molecule that exhibits different mechanisms of action that may be directly related to the inhibition of pathogen’s growth, fertility, and multiplication [28]. The antifungal activity of chitosan was confirmed against phytopathogenic fungi either in vitro or in planta conditions [28]. In regard to *M. fructicola*, the application of chitosan significantly reduced the disease incidence in peach fruits [24], whereas efficient control of postharvest diseases was adequately postulated upon its application [29]. Apart from its fungistatic properties, the elicitation of plant defense responses by chitosan upon facing fungal challenge [19,25,26,27,30,31] allows for its assignment as a promising defense modulator and resistance inducer to combat fungal pathogens, moving towards sustainable disease management.

The detailed transcriptional responses in the peach fruit–chitosan–*M. fructicola* interaction pathosystem remain largely unknown, as well as the putative defense regulatory mechanisms activated in response to chitosan exposure. Here, in order to assess the elicitation potential of chitosan in a compatible interaction between peach and *M. fructicola* and to provide insights into its direct antimicrobial activity against this pathogen through monitoring its transcriptome dynamics, samples were obtained for RNA-seq from fruits treated with chitosan (CHI), fruits inoculated with *M. fructicola* (MF), and fruits treated with both chitosan and the fungus (CHI_MF). Non-treated and non-inoculated fruits, referred to as control fruits (CT), were also analyzed. 

Despite the numerous beneficial properties of chitosan in agriculture, the molecular mechanisms behind its elicitation potential are still unclear. Our results indicate that few DEGs of the CHI-CT group were induced, with almost half of them being suppressed at 12 HAI; their numbers gradually increased at late time points, with even more downregulated DEGs being recorded. Notably, as chitosan has the potential to activate host defense responses, it would be expected to predominantly observe the upregulation of defense-related genes, such as those encoding for pathogenesis-related proteins or enzymes involved in the biosynthesis of secondary metabolites. Instead, activation of a few specific defense-related DEGs was observed mainly at 24 HAI. These findings are consistent with the response of chitosan in sweet oranges where more down than upregulated genes were recorded [18], which was the case as well in chitosan-treated potato leaves where only a few DEGs were induced at early time points, accompanied by the upregulation of only a very limited number of genes directly related to defense [21]. On the contrary, DEGs were highly regulated by chitosan from an early time point and up to one day after treatment in strawberries [4]. Notably, in our study, the defense-related GO term “chitinase activity” was significantly enriched at 48 HAI along with the upregulation of DEGs encoding chitinases, which is in agreement with a previous study [32]. The low number of upregulated defense-associated DEGs may indicate that chitosan induces priming effects in peach fruit in a rather indirect manner, by triggering alterations in gene expression that predominantly target cellular modifications and are not solely or directly involved in pathogen defense, as was also reported in potatoes [21]. Indeed, such cell-mediated defense responses were evident in our study, as the cellular component GO term “cell wall” was only enriched at 12 and 24 HAI, and *KCSs* were constitutively upregulated at these early time points. These genes, which hamper pathogen penetration [33], are associated with priming effects and are involved also in pathways related to fatty acid elongation and plant–microbe interaction [34], which were induced in the CHI-CT group. In addition, for many DEGs associated with the KEGG pathway “cutin, suberine, and wax biosynthesis”, encoding CERs were significantly highly upregulated at early time points upon chitosan treatment. These genes are involved in various stages of wax biosynthesis, carrying out important defense functions [35]. Furthermore, a few CesAs were upregulated at 24 HAI, in contrary with their repression in sweet oranges [18], while two *LRR-EXT* genes were upregulated at 12 HAI. Previously an EXT and a proline-rich cell-wall protein, both involved in cell-wall modifications and hence have potential to participate in defense responses, were upregulated in potato after chitosan treatment [21]. However, as previously postulated [18], other groups of DEGs (*EXPs*, *PLs*, *PMEs*) associated with cell-wall-related pathways were mainly repressed by chitosan treatment at 24 HAI in our study. Their expression profiles indicate that they may play an important role in priming induction, as upregulation of *EXPs* influences cell-wall extensibility and susceptibility [36], whereas *PLs* and *PMEs* are known to promote and mitigate the effect of fruit-ripening-based susceptibility during *B. cinerea* infection through cell-wall loosening [37]. Proteins that loosen the cell wall play a key role in plant vulnerability to pathogen attack [38]. By contrast, we noticed the upregulation of three *CCR* genes at 24 HAI, the activation of which is associated with hampering any upcoming pathogen challenge. 

Unexpectedly, in the CHI-CT group, DEGs related to photosynthesis-associated pathways were significantly upregulated at 24 HAI, whereas this trend was reversed at 48 HAI. The early modulation of such DEGs upon chitosan treatment was also confirmed in strawberry fruits, potatoes, and rice [4,21,39]. As chitosan elicits an apoplastic oxidative burst in many plants [40], this increased photosynthetic activity invariably leads to increased ROS production [4], as is the case in our study. It is also known that priming agents, such as chitosan, trigger redox signaling in order to set plants to an alert state leading to defense responses against prospective pathogen infections [21,41]. Furthermore, this prolonged ROS accumulation may also serve as a direct antimicrobial strategy [21]. However, at some point ROS might be scavenged in order to maintain cellular redox homeostasis through the activation of ROS-responsive regulatory genes. In our study, the GO term “oxidoreductase activity” was revealed to encompass a constitutively significant enriched group across all time points in the CHI-CT group, whereas a *Rboh* (respiratory burst oxidase homolog) gene, which plays an important role in the regulation of ROS accumulation [42], was upregulated at 12 and 24 HAI.

Specific *TF* genes were represented in the expression profile of chitosan-treated fruits, whilst the majority of them were repressed, as in the sweet orange [18]. Notably, among the main TFs associated with defense responses that were modulated with chitosan were two *NAC29* genes that were strongly upregulated early on at 12 HAI, as also recorded in strawberries [4]. Furthermore, two *HY5* genes were upregulated at 24 HAI. Particularly, these TFs play an important role, along with MYCs, a family of bHLH TFs, in the regulation of the JA-mediated activation of defense pathways [43]. In addition, three *TFs* encoding CONSTANS-like proteins with a zinc-finger-binding domain were upregulated at 24 HAI; these genes were previously linked with priming of the tomato’s immune system against *B. cinerea* [44]. In our study, only a few *WRKYs* transcripts were upregulated by chitosan, as was recorded in strawberries [4]. Particularly, we observed an early upregulation of a type of *WRKY15* gene at 12 HAI that influenced disease resistance [45,46], which further indicates that chitosan triggers defense responses in peach fruits within the first few hours after treatment.

Finally, it is worth mentioning that an *LRR-RLK* (*LOC18773395*) gene was significantly upregulated at 12 HAI in the CHI-CT group. Such genes play central roles in signaling upon pathogen perception [47]. On the other hand, the exact recognition mechanism of chitosan in plants is not yet fully clarified, and although it was previously suggested that it can be mediated by chitin receptors, such as CERK [48], in our results the *CERK* gene was not represented in the profile of the CHI-CT group. This suggests that chitosan might be recognized in peach fruits using a CERK independent mechanism, as occurred for the potato [21]. Thus, we are tempted to speculate that this *LRR-RLK* is involved in chitosan perception, possibly triggering a signal cascade involving mitogen-activated protein (MAP) kinases, as previously described for the potato [21].

The expression patterns of our RNA-seq data from the MF-CT group provide further insights into the transcriptome dynamics and the regulatory mechanisms involved in peach fruit responses in a compatible interaction with *M. fructicola*. Our results suggest that peach fruits initiated delayed basal defenses to some extent, but failed to restrict fungal growth and disease progression. This dynamic and time-dependent transcriptional reprogramming upon infection indicates that peach DEGs involved in plant–pathogen interaction, immune-signaling transduction, biosynthesis of secondary metabolites, and other defense responses were triggered no earlier than 24 HAI, and mainly at 48 HAI. Furthermore, the transcriptional profiles of peach fruits coincided with the altered expression of *M. fructicola* genes related to cell-wall-degrading enzymes (CWDEs) and virulence factors, along with the ripening symptoms caused progressively over time by the pathogen.

Breakdown and disassembly of plant cell-wall structure enables pathogen invasion contributing to susceptibility [49], whereas a plant’s response to fungal infection includes various cell-wall-modification processes. Thus, we speculate that cell-wall strengthening predominates among other modifications to impede, to some extent, *M. fructicola* penetration, as previously reported for other necrotrophic fungal infections [50]. Mainly at a late time point, DEGs associated with hampering the pathogen growth including *XTH* and *CesA* genes, which are related to cell-wall thickening and cellulose synthesis were upregulated for the MF-CT group. Such genes were also significantly induced at 48 HAI upon *M. fructicola* infection [51]. *EXT* genes involved in cell-wall reinforcement were constitutively upregulated across the three time points. Moreover, lignin/lignan biosynthesis-related *DIR* genes were upregulated upon *M. fructicola* infection, mainly at 48 HAI. This induction is in agreement with a previous study where a *DIR* gene was detected upon *Monilinia* challenge in peach fruits [52], whereas a *DIR* gene was the most upregulated DEG in unripe strawberry fruits upon *Botrytis* elicitation [49]. As lignin forms a barrier to prevent pathogen penetration and fungal toxin diffusion during the infection of fungal appressoria [53], we assume that lignin biosynthesis genes were partially induced at 48 HAI to boost defense reactions of peach fruits against *M. fructicola* and inhibit microbe-derived degrative enzymes, such as polygalacturonases, cellulases, and glucosidases [54], although it failed to restrict disease progression. Therefore, despite the cell-wall modifications to maintain cell-wall integrity, *M. fructicola* might have manipulated plant cell-wall-degrading enzymes in order to further penetrate the peach fruit tissue. Furthermore, although *EXPs* genes that contribute to cell-wall extensibility and susceptibility to necrotrophic fungi were mostly downregulated at the early time points, they were activated at 48 HAI. Specifically, a late time point upregulation of different cell-wall-degrading DEGs, such as *PGs*, *PMEs*, and different glucosidase-encoding genes, coincides with peach susceptibility to *M. fructicola* as the pathogen directly contributes to cell-wall permeability and tissue softening. 

Early recognition of potential pathogens using PRRs is a crucial step during plant–pathogen interaction in order for plants to respond on time and activate their defense mechanisms [55]. Based on the enrichment analysis of the MF-CT group, the KEGG pathways’ “plant–pathogen interaction”, “MAPK signaling pathway”, and “plant-hormone signal transduction” were significantly induced suggesting that multiple signal transduction pathways were triggered upon *M. fructicola* infection, mainly at the late time. According to our results, the MAPK signaling pathway was activated mainly in the presence of *M. fructicola*, particularly at 48 HAI, suggesting that MAPK induction occurs in the presence of the pathogen. Furthermore, different types of extracellular receptors (RLKs and RLPs) recognized the pathogen’s associated molecular patterns (PAMPs), like fungal CWDEs, and activated PTI, which is the primary line of plant defense. As a result, various membrane-localized DEGs were significantly upregulated across time and were mainly induced at 48 HAI, such as *G*- and *L*-type *LecRK*, *LRR-STK*, *WAK*, and *CRK* genes. Our results are consistent with previous studies where a high number of *RLKs* were upregulated similarly upon *Botrytis* infection [33,49], indicating that these receptors contribute directly in transducing downstream responses. This, hitherto of RLKs’ induction, could regulate immune-signaling pathways and be involved in any defense responses against *M. fructicola* by promoting PTI in peach fruit. It is also worth mentioning that *LRR-STKs* were significantly upregulated at 48 HAI, whereas similar DEGs were previously detected upon *Monilinia* infection [52], as well as upon *M. fructicola* infection on peach fruit [56]. A high repertoire of *TFs* was activated upon *M. fructicola* infection, and the relevant DEGs were progressively upregulated, reaching their highest expression also at 48 HAI. For example, many members of the *WRKY* (*WRKY29*, *WRKY33*, *WRKY45*, *WRKY53*) and *NAC* gene families were highly induced mainly at 48 HAI, indicating their pivotal role as major positive regulators of the defense responses against necrotrophic fungi [57]. On the other hand, high levels of upregulation mainly at 48 HAI of *AP2*/*ERF* genes, which are responsive genes in the ethylene biosynthesis and signaling pathway, indicates their role as susceptibility factors to *M. fructicola* challenge, as was recently reported for different plant–pathogen interaction systems [58,59]. Manipulation of the plant’s HR is a key pathogenicity strategy that necrotrophic fungi have developed [60]. One of the main virulence factors that necrotrophic fungal pathogens exploit to manipulate HR during their colonization is ROS production [61]. Furthermore, lipid peroxidation can be the first indication of a plant’s oxidative-damage response upon a pathogen attack [62]. In our study, lipid-peroxidation and H_2_O_2_ accumulation were significantly increased after *M. fructicola* infection, mainly at 24 and 48 HAI, coinciding with the over-expression of ROS-associated DEGs and severe brown rot symptoms in fruits. Both lipid peroxidation and H_2_O_2_ accumulation were triggered in tomato fruit and citrus leaves after infection with *B. cinerea* and *Alternaria alternata*, respectively [62,63]. Furthermore, DEGs associated with ROS production were significantly induced in nectarine fruit after *M. laxa* infection, as well as in kiwifruit after *B. cinerea* infection [64]. However, the production of ROS scavengers is of utmost importance for plants to keep a balance between damaging cells and to initiate signal transduction and gene expression modulation [65]. In our study, DEGs encoding thioredoxins and glutathione S-transferases were highly upregulated as a result of *M. fructicola* infection.

A key response of plants to modulate defense responses is the production of secondary metabolites, such as terpenoid, phenolic, and flavonoid compounds [53]. In our study, DEGs involved in the flavonoid biosynthesis pathway, like major allergen *Pru ar 1* and *PKS* genes, were highly induced after *M. fructicola* infection. A further biochemical analysis confirmed RNA-seq data, as the concentration of total flavonoids and phenolic compounds was significantly higher compared to the peaches with CT treatment, mainly at 48 HAI. Additionally, in response to *M. fructicola* infection DEGs involved mainly in the phenylpropanoid biosynthesis pathway, which is key to the process of the biosynthesis of phenols, were significantly induced at 48 HAI. Among them, the key enzyme PAL is the first step in phenylpropanoid biosynthesis and contributes to lignin, phytoalexins, and flavonoid biosynthesis [53]. Furthermore, DEGs involved in the flavonoid biosynthesis pathway, such as *PKS*s genes, were also induced in the late stage of infection. Induction of all these DEGs as a response to necrotrophic fungal challenging has already been reported in several studies [49,64]. Furthermore, KEGG pathways related to linolenic acid metabolism were significantly enriched at 48 HAI through the upregulation of *OPRs*, *AOCs*, and linoleate lipoxygenase (*LOX*) genes, reinforcing the hypothesis that their activation is part of a defense repertoire of peach fruit against *M. fructicola*. On the other hand, the high over-expression at 48 HAI of several DEGs encoding ACOs that contribute to the biosynthesis of ethylene might further promote their susceptibility during infection [64]. Thus, although an abundant number of pathogenicity-related genes were significantly upregulated in response to *M. fructicola* infection, mostly at 24 and 48 HAI, it was insufficient to limit fungal expansion. Our results are consistent with several studies where such genes were induced in peach fruit upon infection with the *Monilinia* species, indicating their involvement in brown rot tolerance [51,52].

As a necrotrophic fungus, *M. fructicola* penetrates the fruit surface with the secretion of several CWDEs, such as CAZYmes and hydrolytic enzymes for the degradation of major cell-wall components like cellulose, hemicellulose, and pectin [64]. As expected, a significant number of genes encoding cellulose-degrading enzymes were detected at high levels upon infection, consistent with previous studies where several such genes were detected in *M. fructicola* and *M. laxa* species [66,67]. However, the major carbohydrate of peach fruits is pectin, which requires the production of pectin-degrading enzymes for its breakdown [67]. In our study, *M. fricticola* presented significant high read counts of polygalacturonase 1 (PG1), which is a major pectin-degrading enzyme along with high abundance of genes encoding PME, PNL, GH28, GH53, and CE8, associated with pectin disassembly. Our results are in agreement to previous studies wherein *M. laxa* transcriptome *PG* genes belonging to the GH28 family were highly induced after infection in nectarine fruit [64], whereas after inoculating with *M. laxa* in a liquid medium containing freeze-dried peach, all the above-mentioned pectin-degrading enzymes were secreted [66]. However, some of these enzymes, apart from their pectinolytic activity, are also important virulence factors in necrotrophic fungi [66,68]. We speculate that the significant high abundance of *PG1*, specifically at 48 HAI, concurrent with the high expression of genes associated with a plethora of virulence factors, such as effector proteins, coincides with *M. fructicola* colonization of peach fruit and severe brown rot symptoms at the late stage of infection. We also speculate that the high abundance of genes encoding a CP effector or an NEP protein at the late stage of infection, as well as the effector gene with the CFEM domain at the early stage of infection (12 HAI) might contribute to the cell-death-inducing activity of *M. fructicola* on peach fruits.

Chitosan-induced resistance may be evident in fruits [69,70]. Indeed, DEGs’ expression profile for the CHI_MF-CT comparison group indicates that, although chitosan treatment in CHI-CT group promoted fewer transcriptional changes compared to other treatments, chitosan primes to some extent the activation of the defense mechanisms, but only after *M. fructicola* challenging, which involved primarily reinforcing the cell-wall lignification and the induction of specific defense-related genes, as previously proposed [18,71]. Along the same lines, a commercial formulation of chitosan elicited more pronounced priming defense mechanisms against *B. cinerea* only upon subsequent infection, as chitosan alone did not trigger major changes in transcription with transient or often non-detectable transcriptional reprogramming [44]. Our results highlight that there is a subset of specific DEGs potentially responsible for chitosan-induced priming. Thus, the expression profiles of the CHI_MF-CT group when compared to those of MF-CT revealed that a constitutively higher number of *KCSs*, *CERs*, and *GRPs* genes were upregulated at early time points and mainly at 24 HAI, which indicates that enhanced resistance has been linked with the priming of cell-wall-reinforcing preventing or delaying infection with *M. fructicola*. Furthermore, a smaller number of sensitivity factors, such as beta-glucosidase and *EXPs* DEGs, were constitutively upregulated across all time points for CHI_MF-CT compared to the MF-CT group. A similar trend was also revealed in the case of *PGs* at 48 HAI, which further highlights the cell-wall-related priming effect of chitosan upon the *M. fructicola* challenge. 

The fact that chitosan does not have specific plant receptors [72] may further explain why chitosan does not efficiently activate PTI responses based on the less abundant induction of immune receptors in CHI_MF-CT compared to the MF-CT group. However, it is known that the cell-wall-integrity (CWI)-maintenance mechanism may alternatively trigger defense responses even when PTI signaling is impaired [73]. In this case, the CWI mechanism is monitored through the recognition of oligogalacturonides (OGs), which are products of cell-wall breakdown that are caused by fungal-secreted CWDEs [74]. Furthermore, in the CHI_MF-CT group, less DEGs encoding ABA receptors were upregulated in comparison to the MF-CT group, which further highlights the positive effect of chitosan treatment prior to *M. fructicola* through diminishing the fruit’s ripening. 

Even though the overall *TFs*’ expression profiles in CHI_MF-CT compared to the MF-CT group was quite similar, *ERF*/*AP2* genes, which are the main ethylene response factors, were less induced across the 48 HAI period, indicating once again that a significant smaller number of sensitivity factors were upregulated upon chitosan pre-treatment. In parallel, the expression profiles of *ACO* genes that contribute to the biosynthesis of ethylene revealed also a decrease in their induction in CHI_MF-CT compared to the MF-CT group, which indicates that the expression profiles of specific DEGs might further promote defense responses upon chitosan pre-treatment prior to *M. fructicola* infection. In agreement with our study, it was reported that chitosan induces delayed grapevine defense mechanisms against *B. cinerea* by monitoring the expression of some defense-related genes [27], such as those encoding thaumatin and DEGs involved in JA-mediated signaling (13-lipoxygenase, *LOX13*), in oxylipin pathway (9-lipoxygenase, *LOX9*), and in ethylene biosynthesis (*ACO*). Consistently with our study, chitosan (10 mg/mL) was effective in reducing and delaying disease symptoms of pitch canker in *Pinus patula* leading to reduced lesion lengths [75]. Furthermore, infection of spikelets by *F. graminearum* was slower when wheat spikes were treated with chitosan [76]. Furthermore, chitosan proved to activate host defense in the pea against powdery mildew through induction of the expression of PR proteins, phytoalexins, and lignin synthesis, as well as by enzymes involved in the degradation of the cell wall of pathogens such as chitinase and chitosanase [31]. Based on the effects of chitosan described above, we further investigated physiological indexes in peach fruits to unveil the related mechanisms at the biochemical level. It was obvious that CHI_MF treatment resulted in lower accumulation of H_2_O_2_, and thus less damage to cell membranes (according to the TBARS index), whereas both total phenolic and flavonoid concentrations were lower in peach fruits compared to MF treatment. As in our study, chitosan can act on the phenylpropanoid pathway in tomatoes resulting in the reduction of bacterial spots [77]. 

The direct antimicrobial effect of chitosan against *M. fructicola* was confirmed in our study, as a significantly different expression pattern was observed for the pathogen upon chitosan pre-treatment (CHI_MF) at all time points. Chitosan combines multiple modes of action that are directly related to the suppression of fungal pathogens, which includes the inhibition of mycelial growth and spore germination, restriction of spore movement, changes of hyphal morphology, induction of metabolic disorders, and penetration of the plasma membrane, which triggers the intracellular production of ROS and leads to cell death [11,28,78]. Thus, the gene expression pattern of *M. fructicola* upon chitosan pre-treatment revealed a high number of genes related to metabolism that were not induced in most metabolic pathways compared to the MF treatment. These metabolic disorders may be the root cause of cell death, suggesting that chitosan application directly inhibited the spore germination and mycelial growth of *M. fructicola* [79]. Several genes related to ribosome biogenesis, which determine fungal cell growth, were not triggered by chitosan treatment. These results are in agreement with a previous study where chitosan application at *Aspergillus ochraceus* resulted in the significant downregulation of DEGs involved in ribosome biogenesis [10]. Furthermore, genes associated with lipid, amino acid, and carbohydrate metabolism, which are spore-germination-associated metabolic pathways [80], as well as several sporulation-associated genes were not induced after chitosan pre-treatment. This observation is consistent with previous studies where chitosan application suppressed the expression of genes related to *Phytophthora infestans* sporulation [11]. In addition, genes related to spore-germination-associated proteins like septin and kinesin, ATP-synthases, and proteins required for transcriptional induction [80] were also not induced at any time point during CHI_MF treatment. These results suggest that chitosan directly affected the sporulation and mycelial growth of *M. fructicola* on peach fruits suppressing brown rot symptoms. It is worth mentioning that genes related to the pathogenicity pathways of *M. fructicola*, such as in the production of secondary metabolites (e.g., polyketide syntheses (PKSs), plant CWDEs (e.g., pectin lyase, pectineasterase, cellulase), and biopolymer-degrading CAZymes were not detected or were detected with low read counts, mostly at 48 HAI for CHI_MF treatment, highlighting the direct antimicrobial activity of chitosan. Our results are in agreement with previous studies where chitosan treatment against *P. infestans* [11] and *F. oxysporum* [12] suppressed the expression of their virulence-related genes. Therefore, we suggest that chitosan directly inhibited spore germination and mycelial growth on peach fruits particularly in the early time points of infection. Virulence-related genes of *M. fructicola* were induced mostly with a delay at 48 HAI, coinciding with a delay in the appearance of brown rot symptoms with CHI_MF treatment.

Overall, our results suggest that chitosan not only exhibits direct antifungal properties, but is also able to activate to some extent defense responses in peach fruit before or during the *M. fructicola*-infection process. In the last case, chitosan priming effects are mediated by fine-tuning specific transcriptomic changes, affecting mainly different layers of cell-wall networks that are associated with induced defenses. The proposed roles for these specific genes as primary targets in this modulation of the defense responses, result in the slowing down of the infection process. Our results would allow for better elucidation of peach fruit–chitosan–*M. fructicola* interactions and the design of novel sustainable disease management strategies.

## 4. Materials and Methods

### 4.1. Plant Material, Pathogen Inoculum, and Chitosan Treatment

Intact and mature golden yellow-fleshed peaches (*Prunus persica*, cultivar “O’Henry”) were collected at their ripening developmental stage from an experimental orchard in the Larissa region of central Greece. The fruits were surface-disinfected by dipping them in 5% (*v*/*v*) commercial bleach (10 min), rinsed three times with sterile deionized water, and left to air-dry in a laminar flow hood.

Four treatments were performed: fruits inoculated with *M. fructicola* (MF), fruits treated with chitosan hydrochloride (CHI; Phytorgan SA, Athens, Greece), fruits inoculated with *M. fructicola* and previously pre-treated with chitosan (CHI_MF), and untreated–mock-inoculated control fruits (CT). Fruits with CT and MF treatments were immersed for 1 min in sterile water, whereas fruits containing chitosan applications (CHI and CHI_MF) were immersed in chitosan solution at 1% *w*/*v*, also for 1 min. After 30 min, *M. fructicola* inoculations with MF and CHI_MF fruit treatments were performed using a virulent *M. fructicola* strain (isolate No 2684), which was kindly provided by Benaki Phytopathological Institute. Spore inoculum was prepared by harvesting conidia from 10-day-old PDA cultures, followed by water rinsing, filtered through double gauze and suspending the filtrate in PDB, with the concentration adjusted to 1 × 10^6^ conidia/mL. Inoculations were performed by dropping 40 μL drops of spore suspension or PDB (mock) onto the central adaxial surface of peach fruits. Fruits of CT and CHI treatments were inoculated with 40 μL of sterile water.

The fruits were transferred in sealed transparent containers at 24 °C with high humidity and under standard photoperiod conditions. Fruit tissues were collected by cutting 3 × 3 cm sections of peel and pulp of the peach fruits (four mm deep) around the inoculation sites at 12, 24, and 48 h after inoculation (HAI) in three independent experiments (technical replicates) for all four fruit treatments. Each treatment contained pooled samples from ten fruits. Fruit samples were immediately snap frozen in liquid nitrogen and stored at −80 °C for later use.

### 4.2. Lipid Peroxidation and Hydrogen Peroxide Assays

An amount of 150 mg of fresh plant material (FW) was reduced in fine powder with liquid nitrogen and homogenized with 0.1% trichloroacetic acid (TCA) at 4 °C by vigorous vortexing. After centrifugation at 6500 rpm for 15 min at 4 °C, the supernatant was used for the determination of both lipid peroxidation level and H_2_O_2_ concentration [81]. Briefly, lipid peroxidation was measured as a malondialdehyde (MDA) by-product with a content determined by a reaction with 0.5% 2-thiobarbituric acid (TBA) in 20% TCA (*w*/*v*). For the H_2_O_2_ determination, the reaction mixture consisted of 0.5 mL 0.1% TCA, tissue extract, 0.5 mL of 0.1 M potassium–phosphate buffer (pH 7.0), and 1 mL of 1 M KI (*w*/*v*). The reaction color was measured at 390 nm. Hydrogen peroxide levels were calculated using a calibration curve prepared with eight known concentrations. 

### 4.3. Phenolic and Flavonoid Content on Peach Fruits

The total phenolic (TP) content was estimated using the Folin–Ciocalteu colorimetric method with minor modifications as described in [82]. Briefly, 0.2 mL of the extract diluted with distilled water (1:2) was added into a tube containing 0.2 mL Folin–Ciocalteu reagent and 2.6 mL deionized water. Then, 2 mL Na_2_CO_3_ (7%, *w*/*v*) was added to the mixture, and the absorbance was measured after a 90 min incubation at 750 nm using a spectrophotometer. For TP content, gallic acid was used as the standard reference, and the gallic acid equivalent was expressed as mg per kg of fresh mass. The total flavonoid (TF) content was assayed using the aluminum chloride colorimetric method, while rutin was used as a standard to make the calibration curve. The fruit extract was mixed with 0.2 mL of 10% (*w*/*v*) AlCl3 solution in acetone, 0.2 mL potassium acetate (1 M), and 5.6 mL water. The mixture was incubated for 30 min at room temperature, followed with the measurement of absorbance at 415 nm. For TF content, rutin was used as the standard reference, and the rutin equivalent was expressed as μg per g of fresh mass. 

### 4.4. Transcriptome Sequencing

RNA-seq was performed for the fruit samples of all four treatments collected at 12, 24, and 48 HAI across the three biological replicates. Thus, the total RNA of each sample out of the 36 in total was isolated employing the Monarch Total RNA Miniprep Kit (NEB, Frankfurt, Germany). Sequencing libraries were constructed using the PT042 NGS RNA Library Prep Set (Novogene Ltd., Cambridge, UK) and were sequenced on the Illumina Novaseq 6000 platform generating 2 × 150 bp (paired-end) reads. Raw sequencing data are available at Sequence Read Archive (SRA) database under the BioProject accession number PRJNA1035538.

### 4.5. Sequence Mapping Analysis

Raw data were processed with fastp v0.23.4 software, and clean reads were obtained by removing reads containing adapters, poly-N sequences, and low-quality reads. The reference peach genome and the gene models data of cultivar Lovell [83] were downloaded from the NCBI genome database. The clean paired-end reads were mapped to the reference genome, employing the HISAT2 software (v2.0.5.) [84]. Gene expression levels were calculated using the FPKM method and differentially expressed genes (DEGs) were assigned, employing the DESeq2 R package (1.20.0) based on the negative binomial distribution [85]. DEGs were assigned according to an absolute value of log2 fold change ≥ 1 and an adjusted *p*-value ≤ 0.05 using the Benjamini and Hochberg’s approach for checking the false discovery rate (FDR). In order to detect *M. fructicola* transcripts, reads belonging to its transcriptome were identified from the inoculated samples (MF and CHI_MF treatments) at their respective sampling times. Toward this end, the unmapped peach genome reads from these samples were mapped to the *M. fructicola* strain Mfrc123 genome assembly (ASM869222v1) using the HISAT2 software [84]. The assignment of reads on a transcript level was performed employing the htseq-count program, and the normalized transcript counts were calculated using the DESeq2 R v1.20.0 software [85].

### 4.6. Functional Annotation

Gene ontology (GO) enrichment analysis was implemented using clusterProfiler R package (3.8.1), in which gene length bias was corrected [86], where GO terms with a *p*-value < 0.05 were considered significantly enriched by DEGs. The KOBAS v3.0 software was used to assign KEGG Orthology terms on DEGs, and clusterProfiler R package (3.8.1) was used to test the statistical enrichment of DEGs in KEGG pathways.

### 4.7. Gene Expression Validation

To validate the RNA-seq data, real-time RT-PCR for gene expression quantification of several peach DEGs was performed. Aliquots were taken from the same RNAs that were used for sequencing and first-strand cDNA was synthesized using the LunaScript^®^ RT SuperMix Kit (NEB, Europe) according to the manufacturer’s instructions. The expression analysis was performed using the Luna^®^ Universal qPCR Master Mix (NEB, Europe) with PCR parameters as recommended by the manufacturer at QuantStudio^®^ 5 Real-Time PCR System (Applied Biosystems, Foster City, CA, USA). PCR reactions were completed in triplicate. The expression profiles of nine randomly selected DEGs were analyzed and normalized through comparison with a reference housekeeping gene (LOC18789459). Relative quantitative expression ratios of inoculated samples compared to respective controls were calculated according to the 2^−ΔΔCT^ method [87] using three technical replicates for each DEG evaluation. All gene-specific primers are listed in Table S10.

## 5. Conclusions

In conclusion, the present study shows that chitosan could effectively control brown rot in peach fruit by delaying the hyphal development of *M. fructicola* and triggering priming-related defense responses during infection when used as a pre-inoculant. These responses at transcript level based on our RNA-seq approach were unveiled to deeply modulate mainly cell-wall-modification dynamic processes that induce fine-tuned changes in the kind, quantity, and timing of specific genes involved in chitosan-induced tolerance. Thus, chitosan elicitor seems to regulate the expression patterns of specific sensitivity factors and defense-related genes driving the response network in the early phase after its application. Meanwhile, we have here highlighted that during the compatible peach fruit–*M. fructicola* interaction expression patterns of specific DEGs promote susceptibility to infection and trigger delayed defense responses, suppressing pathogen growth to some extent, although it failed to restrict disease progression. Notably, the gene expression patterns of *M. fructicola* revealed that chitosan exhibits direct antifungal activity by inhibiting the induction of genes involved in spore germination, mycelial growth, metabolic and pathogenicity pathways, and diminishing the pathogen virulence. As this study provides insights into how chitosan might provide protection by directly acting as an antifungal agent or stimulating peach defense responses to *M. fructicola* infection, we suggest that the application of chitosan could be an effective and promising approach to control brown rot in peach fruit.

## Data Availability

The datasets generated during the current study are available in the NCBI SRA database below: https://www.ncbi.nlm.nih.gov/, PRJNA1035538, created date 3 November 2023.

## Supplementary Materials

The following supporting information can be downloaded at https://www.mdpi.com/article/10.3390/plants13050567/s1, Figure S1: Volcano plots of differentially expressed genes between CHI, MF, CHI_MF, and CT treatments at three time points; Figure S2: Venn diagram representing the distribution of DEGs across the four treatments at three time points; Figure S3: gene ontology-based functional categorization of the most representative differentially expressed genes; Figure S4: hierarchical clustering of differential expressed genes of *M. fructicola* based on the average counts of normalized reads between MF and CHI_MF treatments versus CT treatment at three time points; Figure S5: comparison of RNA-seq and RT-qPCR expression values of selected genes in three different time points for peach fruit after *M. fructicola* infection; Table S1: summary of peach fruits RNA-seq data; Table S2: *M. fructicola* expressed genes and average counts of normalized reads among the three replicates of *M. fructicola*-inoculated peach samples, either individually (MF) or in combination with chitosan (CHI_MF) at three time points (12, 24 and 48 HAI); Table S3: DEGs profiles in peach fruits among three different comparison groups (CHI–CT, MF–CT, CHI_MF–CT) at 12, 24, and 48 HAI; Table S4: DEGs involved in cell-wall modification; Table S5: DEGs involved in pathogen perception and signaling transduction; Table S6: DEGs encoding TFs; Table S7: DEGs encoding pathogenesis-related and defense proteins; Table S8: DEGs involved in secondary and primary metabolism; Table S9: DEGs encoding nutrient and ion transporters; Table S10: Specific primers used in qRT-PCR for the validation of RNA-seq data.
