# Supplementary material for: Global Transcriptome Analysis of the Peach (Prunus persica) in the Interaction System of Fruit–Chitosan–Monilinia fructicola
Source: Plants (Basel). 2024 Feb 20;13(5):567. doi: 10.3390/plants13050567 (PMC10933875; doi:10.3390/plants13050567)
Supplement: Supplementary file 1 [file plants-13-00567-s001.zip › Tsalgatidou_et_al_2024_Supplementary_Figures.pdf]

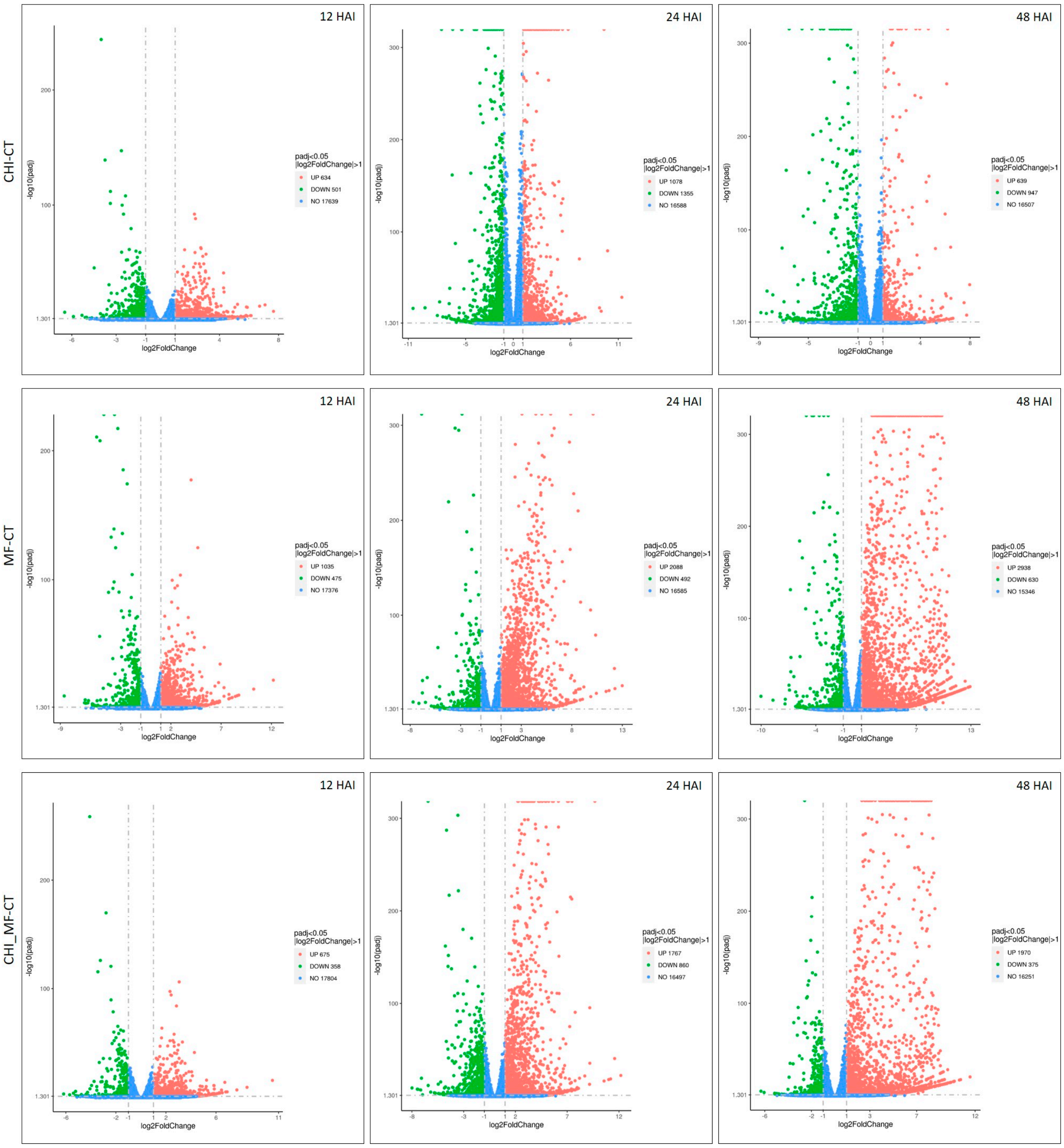

Figure S1. Volcano plots of differential expressed genes between CHI, MF, CHI_MF and CT treatments at three time points. The green part represents downregulated genes and the red part represents up-regulated genes. No significantly expressed genes are represented in blue. The x-axis represents the log2 (fold change) and the y-axis the base mean expression values.

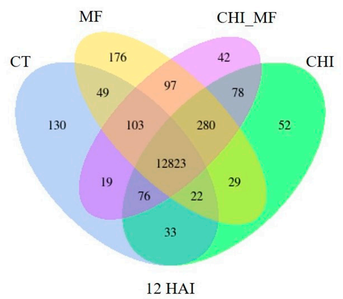

Figure S2. Venn diagram representing the distribution of DEGs across the four treatments at three time points.

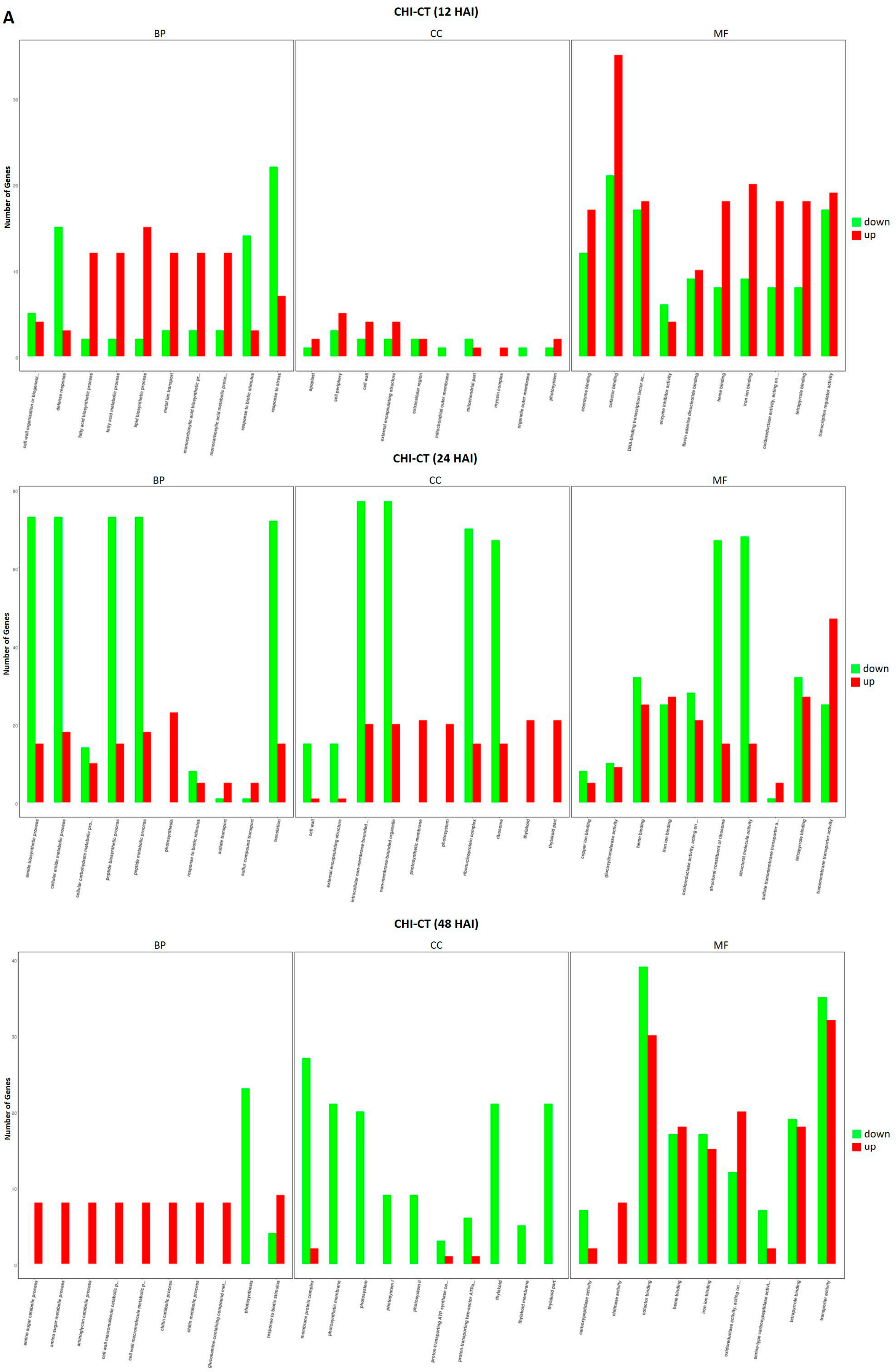
A
CHI-CT (12 HAI)
CHI-CT (24 HAI)
CHI-CT (48 HAI)
BP
CC
MF
Number of Genes
down
up

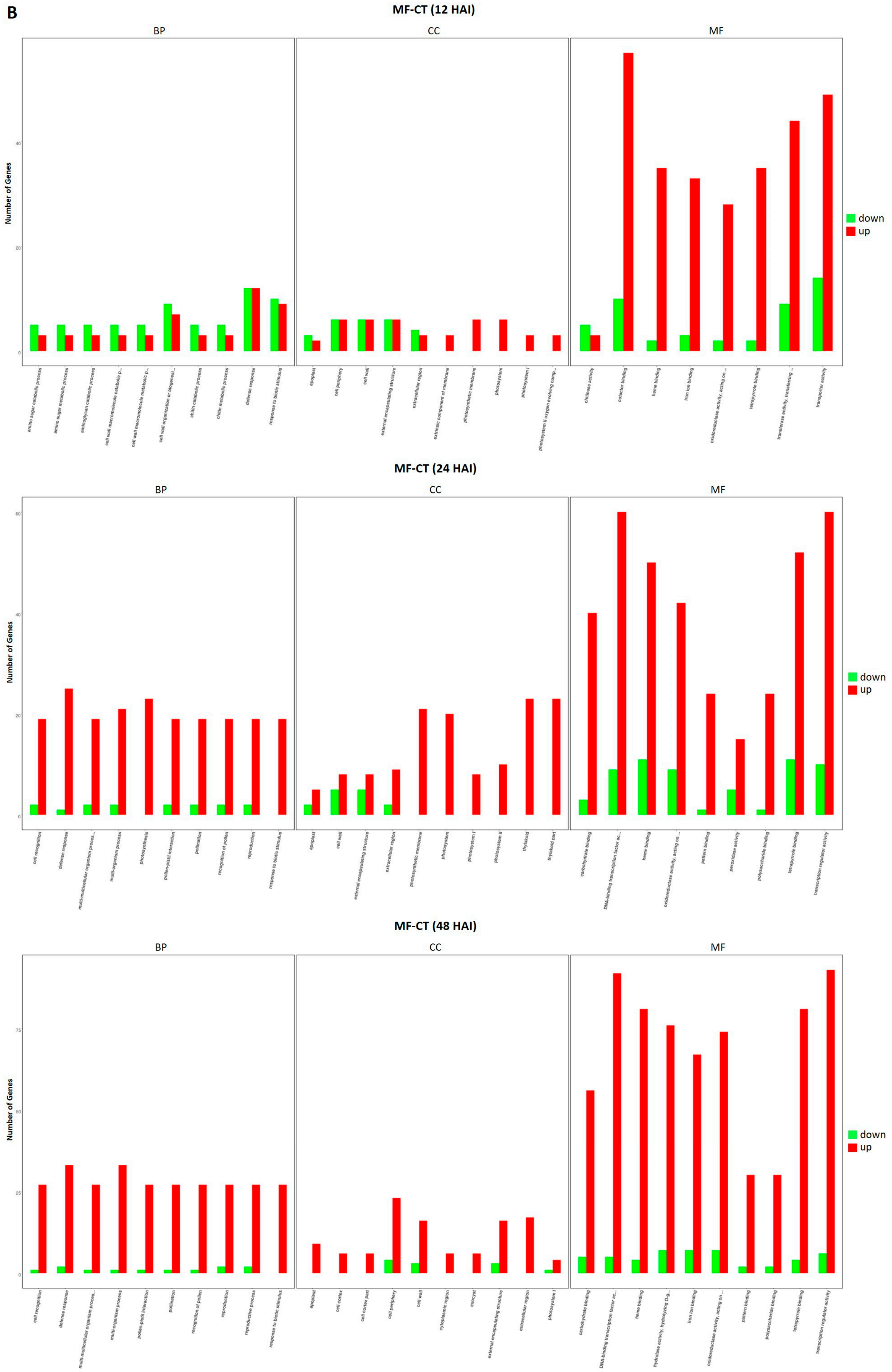
B
MF-CT (12 HAI)
BP
CC
MF
Number of Genes
down
up
MF-CT (24 HAI)
BP
CC
MF
Number of Genes
down
up
MF-CT (48 HAI)
BP
CC
MF
Number of Genes
down
up

C

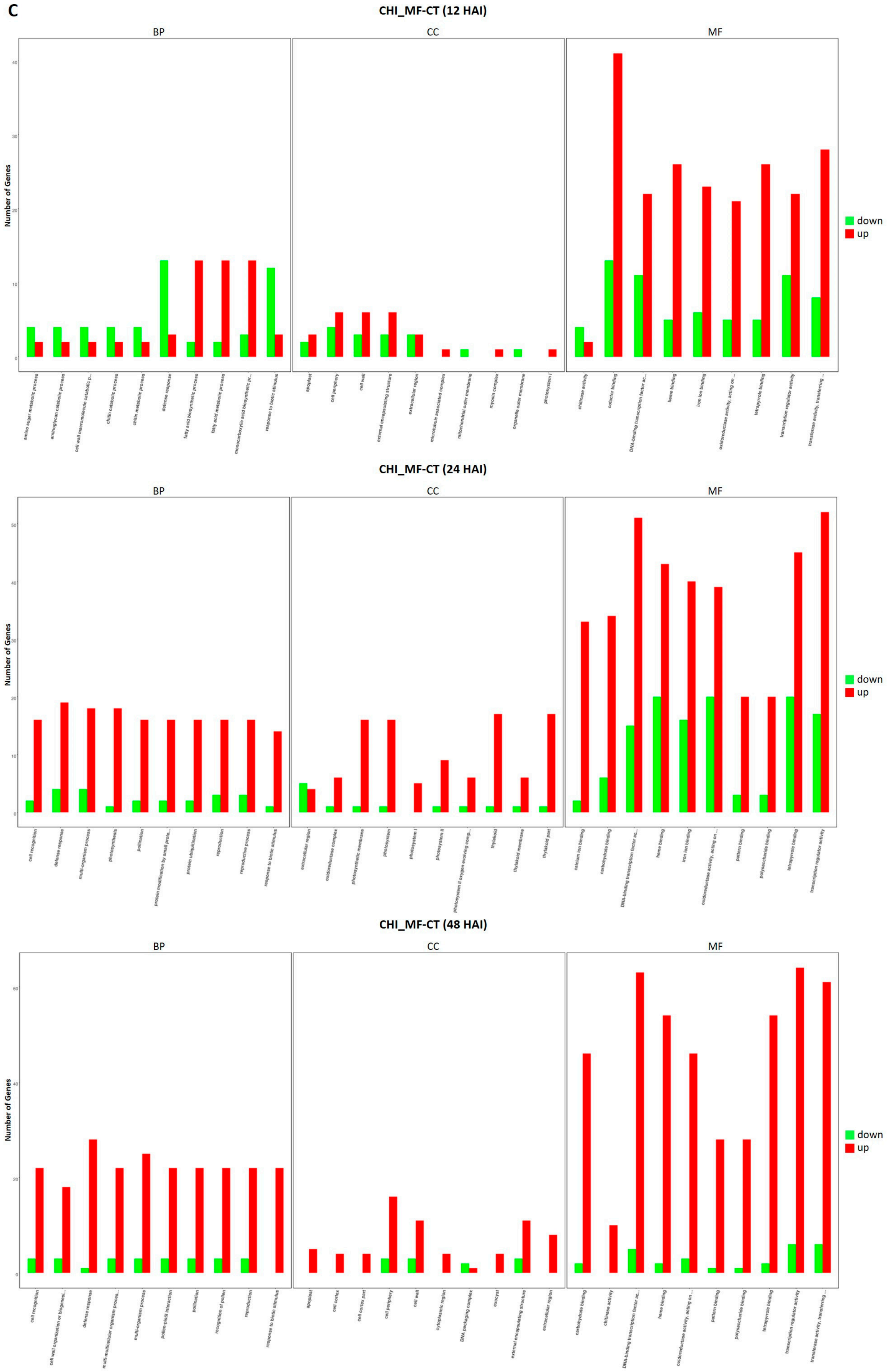

Figure S3. Gene Ontology-based functional categorization of the most representative differentially expressed genes. The red and green bars indicate the number of up-and down-regulated genes, respectively, between A) chitosan (CHI) treated and control (CT) fruits, B) *M. fructicola* (MF)

inoculated and control (CT) fruits, and C) chitosan pre-treated - *M. fructicola* inoculated (CHI_MF) and control (CT) fruits, at three time points.

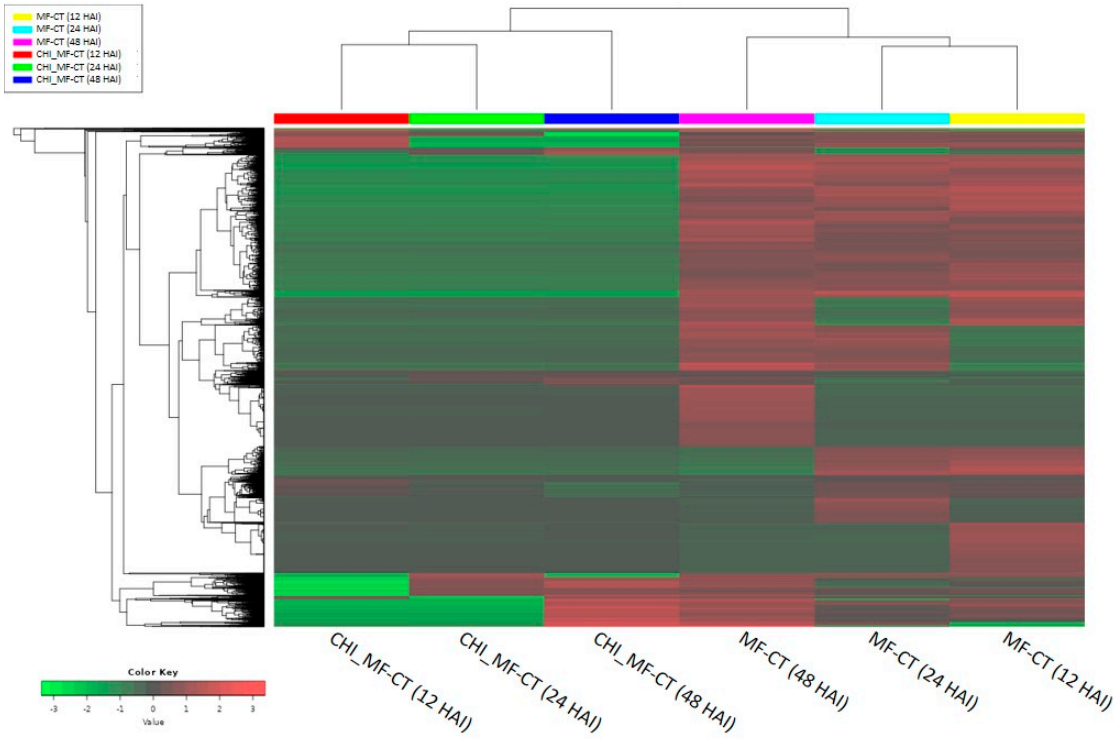

Figure S4. Hierarchical clustering of differential expressed genes of M. fructicola based on the average counts of normalized reads between MF and CHI_MF treatments versus CT treatment, at three time points. Green and red colors in each node of the dendrogram represent repressed and induced, respectively, expression of *M. fructicola* genes.

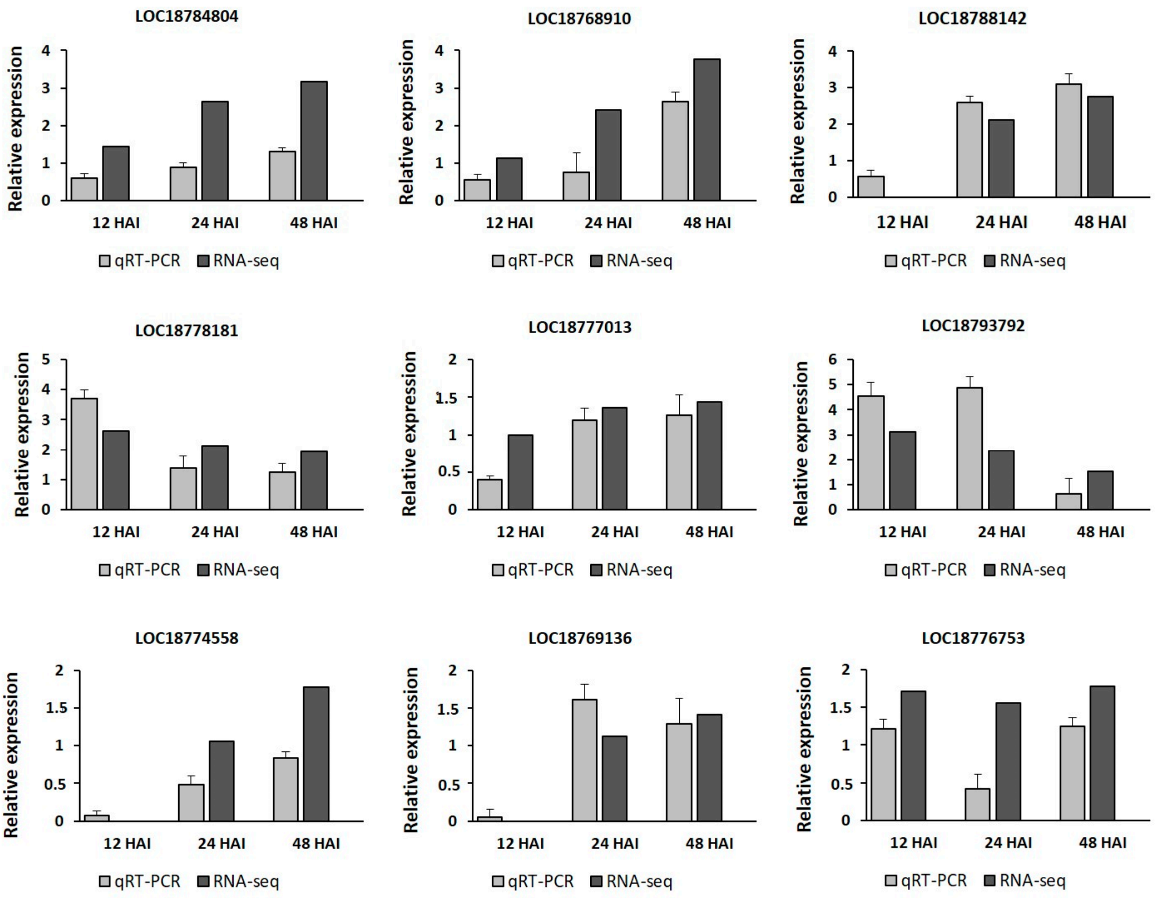

Figure S5. Comparison of RNA-seq and RT-qPCR expression values of selected genes in three different time-points of peach fruit after *M. fructicola* infection. RNA-seq expression levels are depicted as log2 fold change values. RT-qPCR expression levels are represented as mean values from three independent biological replicates.
